# Editorial: Perspectives in Small Animal Radionuclide Imaging

**DOI:** 10.3389/fmed.2020.00262

**Published:** 2020-06-16

**Authors:** Francesco Cicone, Gaurav Malviya, Gianmario Sambuceti

**Affiliations:** ^1^Unit of Nuclear Medicine, Department of Experimental and Clinical Medicine, “Magna Graecia” University of Catanzaro, Catanzaro, Italy; ^2^Department of Nuclear Medicine and Molecular Imaging, Lausanne University Hospital, Lausanne, Switzerland; ^3^Translational Molecular Imaging, Cancer Research UK Beatson Institute, Glasgow, United Kingdom; ^4^Nuclear Medicine, IRCCS Ospedale Policlinico San Martino, Genoa, Italy

**Keywords:** small animal models, radionuclide imaging, molecular imaging, translational research, micro-PET imaging, micro-SPECT imaging

The development of preclinical tomographic imaging techniques for photon detection, either single (micro-SPECT), or in coincidence (micro-PET), has demonstrated an incredible potential in the field of molecular imaging using genetically modified animal models of different diseases. The coupling of small animal PET and SPECT scanners with computed tomography (CT) or magnetic resonance (MR) has fully reproduced the hybrid imaging tools available at human scale. Radionuclide imaging allows non-invasive longitudinal assessment of biological processes, such as angiogenesis, hypoxia, metabolic heterogeneity, cell proliferation, receptor dynamics, and monitoring response to therapy at the molecular level. Additionally, these preclinical imaging tools are designed help in the early stage research of novel therapeutics to provide proof of concept for target modulation and other underlying processes that can help in decision making and may explain or predict therapy outcomes. At present, the spectrum of potential targets for imaging and therapy has dramatically broadened, and testing on small animal models has become a standard in modern medicine. The use of small animal models requires deep understanding of the biological consequences introduced by the genetic manipulations and by the experimental conditions, as well as of the intrinsic interspecies diversities. These aspects are crucial to the interpretation of the data generated in small animals and to a successful translation to bedside.

A focused Research Topic was hosted by the section of Nuclear Medicine of *Frontiers in Medicine* with the aim of covering some of the numerous interdisciplinary features of small animal radionuclide imaging. These include new technological advancements, development of innovative drugs, and translational aspects of animal models of diseases.

Five articles were published in the Research Topic between November 2018 and May 2019. Two of these articles concerned recent developments of micro-PET technology, while three additional papers discussed the relevance of small animals models for translational research for imaging and therapy of human diseases.

Gonzalez et al. designed a micro-PET insert compatible with high-field MR, based on a single LYSO crystal with a cylindrical inner shape and a polygonal external configuration. This setup would be able to circumvent the significant degradation of spatial and energy resolution occurring at the edges of standard scintillation crystals based on individual modules. The authors proved their concept by performing a series of simulations based on experimental data acquired with available technology.

Molinos et al. reported on the performances of the integrated microPET/CT system recently developed by Bruker Biospin GmbH (Ettlingen, Germany), with particular regard to dose optimization. In fact, limiting the absorbed doses to small animals by keeping a satisfactory image quality is a challenge for preclinical imaging. To this purpose, the developed technology takes advantage of the high sensitivity and resolution of a PET subsystem based on silicon photomultipliers, as well as of the fast yet highly performing CT component.

The short review written by Donche et al. was dedicated to radiation therapy for glioblastoma in small animals. The authors introduced the principles of PET-based dose painting and provided a nice summary of the preclinical research platforms that were developed worldwide to integrate image-based dose planning into external beam radiation therapy protocols in small animals. Potentials and pitfalls of the implementation of either optical imaging or PET in radiotherapy planning of small animals were also discussed.

The article of Meester et al. covers many aspects concerning imaging atherosclerosis in small animals. The extremely dynamic environment of atherosclerotic plaques is one of those biological contexts where radionuclide imaging is particularly advantageous over purely morphological imaging techniques. This translates into a plethora of available radiopharmaceuticals, targeting different components and processes occurring around atherosclerotic plaque formation. Mouse models of atherosclerosis are well-established, although the differences of plaque composition and stability between transgenic mice and real patients should always be taken into account. The authors also discuss technical aspects related to the hardware design of preclinical SPECT and PET cameras, and their relevance for the imaging of atherosclerotic plaques.

The article of Bouter and Bouter offers a comprehensive review of the available transgenic mouse models of Alzheimer's disease (AD), and of the results of ^18^F-fluorodeoxyglucose (FDG) in these models as compared to wild-type animals. FGD PET is expected to be useful for the longitudinal assessment of mouse models of AD, particularly for the evaluation of the effects of new therapies on brain function. However, the study of brain glucose metabolism in mouse models of AD gave contradictory results, which could be explained by the methodological heterogeneity between studies or by the biological differences between these models and their human counterpart.

In summary, the articles published in this Research Topic provide an overview of recent developments and perspectives of small animal radionuclide imaging, both from a technical and a biological point of view. We think that our efforts as guest editors have helped to raise the awareness of the readers of *Frontiers in Medicine* about the expanding contributions that preclinical radionuclide imaging offers to biological research. We strongly encourage the editorial board to continue soliciting scholars to submit their innovative small animal radionuclide imaging research findings on the dynamic platform of *Frontiers in Medicine*.

## Author Contributions

FC, GM, and GS wrote and approved the final version of the manuscript.

## Conflict of Interest

The authors declare that the research was conducted in the absence of any commercial or financial relationships that could be construed as a potential conflict of interest.

